# Trends in age‐sex‐specific prevalence and incidence of antidepressant dispensation in the Nordic countries: a systematic review

**DOI:** 10.1002/bcp.70540

**Published:** 2026-03-24

**Authors:** Marcel Ballin, Paulina Tuvendal, Carola Bardage, Mats Persson, Mats Talbäck, Rickard Ljung

**Affiliations:** ^1^ Division of Use and Information Swedish Medical Products Agency Uppsala Sweden; ^2^ Department of Public Health and Caring Sciences, Clinical Geriatrics Uppsala Universitet Uppsala Sweden; ^3^ Institute of Environmental Medicine Karolinska Institutet Stockholm Sweden

**Keywords:** anxiety, depression, drug, pharmacotherapy, prescription, psychiatric, treatment

## Abstract

Differences in antidepressant dispensation across the Nordic countries have been reported, but existing reviews are based on older data. We conducted a systematic review of population‐based studies reporting prevalence and/or incidence of antidepressant dispensation in the Nordic countries. Embase, PubMed and Web of Science were searched for studies published between 1 January 2000 and 1 October 2025. Of 2728 screened records, 30 studies with data from 1994 to 2021 were included. Comparisons with Finland and Iceland were not possible due to outdated data. Antidepressant dispensation rates were higher in Sweden than in Denmark and Norway, with differences increasing over time, largely driven by rising rates in Sweden. This pattern was observed across age groups and sexes but was most pronounced among children and adolescents, particularly girls. The most recent data showed approximately 2.0–5.6 times higher prevalence and incidence of antidepressant dispensation in Sweden compared with Denmark and Norway. In 2021, the prevalence per 1000 population among 15‐ to 19‐year‐olds was 87.9 for girls and 34.7 for boys in Sweden, compared with 35.8 for girls and 14.5 for boys in Denmark and 31.6 for girls and 12.5 for boys in Norway, with a similar pattern for incidence. For adults, the prevalence pattern was similar, although incidence data were lacking. These findings underscore the need for analytical studies examining factors underlying temporal trends and cross‐national differences in antidepressant dispensation, including incidence trends among adults, as well as updated population‐based studies from Finland and Iceland to enable comprehensive Nordic comparisons.

## 1 INTRODUCTION

Psychiatric disorders, such as depressive, anxiety and obsessive‐compulsive disorders, account for a large proportion of global disease burden and are associated with reduced life expectancy and significant economic burden.^1^, ^2^, ^3^, ^4^ The Nordic countries, which consist of Denmark, Finland, Iceland, Norway and Sweden, have a high psychiatric morbidity and mortality burden,^1^, ^4^, ^5^ as well as a high consumption of antidepressive medications used in the treatment of several of these disorders.^6^ There are many similarities between the Nordic countries, such as comparable populations, healthcare and drug reimbursement systems,^7^ and prevalence of depressive and anxiety disorders. A recent study estimated that the prevalence of major depression in specialized care was 3.1% in Sweden, 3.2% in Denmark and 4.4% in Norway,^5^ with comparable findings reported also for anxiety disorders.^8^ At the same time, emerging evidence indicates increased variations across the countries in the dispensation of antidepressant medications.^9^

A recent systematic review of antidepressant dispensation in the Nordic countries found a much higher level of dispensation in Sweden compared to Denmark and Norway.^9^ However, the review focused on children and adolescents and was based on older data, as the most recent prevalence data were from 2017 and the most recent incidence data from 2013. Whether the gap in dispensation may have narrowed or even widened in recent years remains unclear, but it may be hypothesised that dispensation could have continued to increase in recent years,^10^ alongside a worsening in mental health.^11^ An updated review of contemporary, population‐wide evidence, especially with age‐ and sex‐specific estimates, could yield valuable insights on recent trends. Therefore, we conducted a systematic review of the prevalence and incidence of antidepressant dispensation across all age groups in the Nordic countries, with a focus on time trends in age‐sex specific estimates.

## 2 METHODS

This systematic review was conducted within the scope of a government investigation targeting antidepressant prescribing in Sweden compared to the other Nordic countries, which was assigned to the Swedish Medical Products Agency on 16 December 2024 on behalf of the Government Offices of Sweden, Ministry of Social Affairs. The protocol for this review was pre‐registered (Prospero CRD420250651803),^12^ and the report follows the PRISMA guidelines.^13^ Ethical approval was not required because only previously and publicly available data were used in this study.

### 2.1 Eligibility criteria

Eligible studies were observational studies written in English or Scandinavian languages (i.e., Danish, Norwegian and Swedish), published since 2000 and reporting yearly prevalences and/or incidences of antidepressant prescribing in the general population in the Nordic countries, with antidepressant prescribing defined using Anatomical Therapeutic Chemical classification system code N06A. In the Nordic countries, there are nationwide drug registries that contains information on all drugs dispensed at pharmacies.^7^ Despite the publication cut‐off of year 2000, we considered studies spanning data across the 1990s and 2000s for inclusion, in order to not miss potentially relevant and important articles. We excluded studies meeting any of the following criteria: (i) not focusing on antidepressant prevalence/incidence per se, (ii) conducted in non‐population‐based samples (e.g., clinical samples), (iii) only reporting cumulative estimates and (iv) relying on self‐reported drug use.

### 2.2 Literature search strategy

An information specialist at the Swedish Medical Products Agency developed the literature search strategy (Supporting Information S1 and S2) in collaboration with three reviewers (MB, PT and RL). Literature searches were carried out in Embase, PubMed and Web of Science, searching for articles published until 4 April 2025, with an updated search for articles published until 1 October 2025, using keywords like ‘antidepressants’, ‘prevalence’ and ‘incidence’. Both free text terms and controlled terms were used. Boolean operators were applied to combine terms, and proximity operators were used when appropriate. The literature search was supplemented by backward and forward citation searching of included articles.

### 2.3 Study selection, data extraction and presentation of results

The articles were imported into Rayyan systematic review software.^14^ After duplicates removal, one reviewer (MB) screened all articles at the title level and two reviewers (MB and PT) independently screened all remaining articles at the abstract and full‐text level. Disagreements were discussed, and conflicts were resolved by consulting a third reviewer (RL), until consensus was reached. Data extraction of all included studies was performed by one reviewer (MB) and double‐checked by a second reviewer (MT). The data were extracted into Microsoft Excel and included bibliometric information, study characteristics (i.e., study period, country, population size, age, proportion of female participants and antidepressant definition) and outcome data (i.e., yearly prevalence and/or incidence of dispensed antidepressants). We contacted the authors of articles in which relevant data were not sufficiently reported, such as age‐sex specific estimates.

Estimates across studies were harmonized to be expressed as proportions per 1000 population per person‐years. We also quantified relative differences by calculating ratios of estimated values between countries at the most recent time point.

### 2.4 Study quality

Study quality of all included studies was assessed independently by two reviewers (MB and PT) using the Joanna Briggs Institute Critical Appraisal Checklist for Studies Reporting Prevalence Data.^15^ This tool assigns a score ranging from 0 (*lowest quality*) to 9 (*highest quality*) based on the following domains: appropriateness of the sample frame; recruitment procedure; adequacy of the sample size; description of participants and setting; coverage of the identified sample; validity of the method used to identify antidepressants; reliability of the method used to identify antidepressant; adequacy of statistical analyses; and response rate. One point per domain is assigned.

## 3 RESULTS

### 3.1 Study selection

**FIGURE 1:**
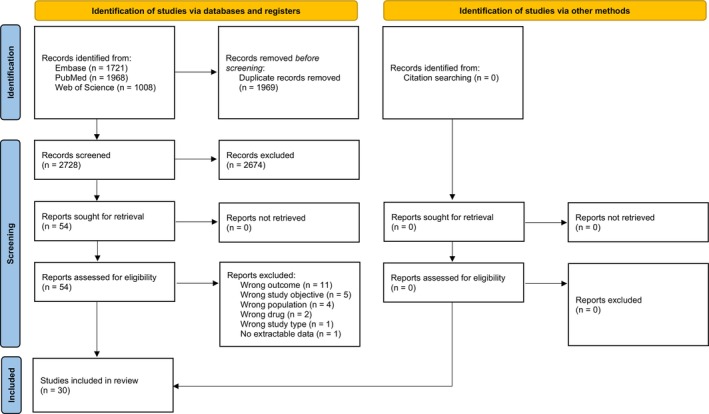
PRISMA flowchart of the study selection.

The literature search generated a total of 4697 records. After duplicates removal, 2728 unique articles remained and were screened at the title and abstract level against eligibility criteria. Of these, 54 were subject to screening at full‐text level. From these articles, 24 were excluded (Table S1), whereas 30 met the inclusion criteria. Backward and forward citation searching did not yield additional articles. Thus, a total of 30 articles were included in the review (Figure 1).

### 3.2 Study characteristics and quality

The 30 studies (Table 1) reported estimates from 1994 to 2021 and covered Denmark (*n* = 13, of which 1 also included Greenland), Finland (*n* = 5), Iceland (*n* = 1), Norway (*n* = 10) and Sweden (*n* = 9).^16^, ^17^, ^18^, ^19^, ^20^, ^21^, ^22^, ^23^, ^24^, ^25^, ^26^, ^27^, ^28^, ^29^, ^30^, ^31^, ^32^, ^33^, ^34^, ^35^, ^36^, ^37^, ^38^, ^39^, ^40^, ^41^, ^42^, ^43^, ^44^, ^45^ Prevalence of dispensed antidepressants was reported in 26 studies and incidence in 11 studies. The definition of prevalence was similar across studies, whereas the definition of incidence varied, with wash‐out periods ranging from no previous dispensation to 3 years and 1 year being the most commonly used. Ages in the studies varied and sometimes comprised both children and adults, but almost half of the studies (*n* = 14) exclusively targeted people aged <20 years, whereas almost a quarter (*n* = 7) focused on adults. Twenty‐nine studies used large‐scale nationwide or regional prescription/medical databases, whereas one included review of medical records. Twenty‐eight considered all antidepressants, whereas two studies were restricted to selective serotonin reuptake inhibitors.

**TABLE 1:** Study characteristics of included studies.

Study	Setting/context	Country	Study period	Sample size	Ages	Female, %	Types of antidepressants	Measures	Prevalence definition	Incidence definition
Abbing‐Karahagopian 2014	Nationwide prescription registry	Denmark	2001–2009	Not specified	All	Not specified	All antidepressants	Prevalence	Number of patients with at least one dispensation in a calendar year divided by the total number of person‐years in the same calendar year	NA
Autti‐Rämö 2011	Nationwide prescription registry	Finland	1997–2007	1.7 million	0–26	48.9	All antidepressants	Prevalence and incidence	Number of patients with at least one dispensation in a calendar year divided by the total population within the same calendar year.	Number of patients with at least one dispensation in a calendar year, and no dispensation during the past 1 year, divided by the total number of inhabitants within that calendar year.
Bachmann 2016	Nationwide prescription registry	Denmark	2005–2012	1.2 million	0–19	48.7	All antidepressants	Prevalence	Number of patients with at least one dispensation in a calendar year divided by the total population within the same calendar year.	NA
Bojanić 2024	Nationwide prescription registries	Denmark, Norway, Sweden	2006–2021	Not specified	All	Not specified	All antidepressants	Prevalence	Number of patients with at least one dispensation in a calendar year divided by the total population within the same calendar year.	NA
Corneliusson 2024	Population‐based clinical study	Sweden	2000–2017	1611	85 and older	60.9 to 82.8	All antidepressants	Prevalence	Number of patients with at least one prescription in a calendar year divided by the total population within the same calendar year.	NA
Forslund 2020	Regional administrative health care data	Sweden	2007–2017	1.7 million	20 and older	Not specified	All antidepressants excluding amitriptylin	Prevalence	Number of patients with at least one dispensation in a calendar year divided by the total study population within the same calendar year.	NA
Foulon 2010	Nationwide prescription registry	Finland	1998–2005	Not specified	0–19	Not specified	All antidepressants	Prevalence and incidence	NA	Number of patients with at least one dispensation in a calendar year, and no dispensation during the past 6 months, divided by the total number of inhabitants within the same calendar year.
Gómez‐Lumbreras 2021	Nationwide prescription registries	Denmark, Norway, Sweden	2008–2017	Not specified	0–19	Not specified	All antidepressants	Prevalence	Number of patients with at least one dispensation in a calendar year divided by the total study population within the same calendar year.	NA
Hansen 2007	Regional prescription database	Denmark	1992–2004	0.5 million	20 and older	Not specified	All antidepressants	Prevalence	Number of patients with at least one dispensation in a calendar year divided by the total study population within the same calendar year.	NA
Hartz 2016a	Nationwide prescription registry	Norway	2004–2013	Not specified	13–17	Not specified	All antidepressants	Prevalence and incidence	Number of patients with at least one dispensation in a calendar year divided by the total population within the same calendar year.	Number of patients with at least one dispensation in a calendar year, and no dispensation during the past 3 years, divided by the total population within the same calendar year minus the number of prevalent users during the past 3 years.
Hartz 2016b	Nationwide prescription registry	Norway	2004–2014	1.1 million	0–17	48.8	All antidepressants	Prevalence	Number of patients with at least one dispensation in a calendar year divided by the total population within the same calendar year.	NA
Ingemann 2021	Population‐based and nationwide medical registers	Denmark, including Greenland	2019	5.2 million	10–89	50.3	All antidepressants	Prevalence	Number of patients with at least one dispensation in a calendar year divided by the total study population within the same calendar year.	NA
Ishtiak‐Ahmed 2023	Nationwide prescription registry	Denmark	2015–2019	1.2 million	65 and older	About 54	All antidepressants	Incidence	NA	Number of patients, including prevalent users, with at least one new dispensation in a calendar year, divided by the total number of person‐years within the same calendar year.
Kjosavik 2011	Nationwide prescription registry	Norway	2004–2009	4.8 million	All	Not specified	All antidepressants	Incidence	NA	Number of patients with at least one dispensation in a calendar year, and no previous dispensation, divided by the total study population within the same calendar year.
Kjosavik 2009	Nationwide prescription registry	Norway	2005	4.6 million	All	Not specified	All antidepressants	Prevalence	Number of patients with at least one dispensation in a calendar year divided by the total study population within the same calendar year.	NA
Lagerberg 2019	Nationwide prescription registry	Sweden	2006–2013	Not specified	0–24	Not specified	All antidepressants	Prevalence	Number of patients with at least one dispensation in a calendar year divided by the total study population within the same calendar year.	NA
Lien 2023	Population‐based survey linked to prescription registry	Norway	2004–2020	0.1 million	15–19	About 50	All antidepressants	Prevalence	Number of patients with at least one dispensation in a calendar year divided by the total study population within the same calendar year.	NA
Loikas 2013	Nationwide prescription registry	Sweden	2010	9.3 million	All	Not specified	All antidepressants	Prevalence and incidence	Number of patients with at least one dispensation in a calendar year divided by the total study population within the same calendar year.	Number of patients with at least one dispensation in a calendar year, and no dispensation during the past 1 year, divided by the total number of person‐years within the same calendar year.
Pottegård 2014	Nationwide prescription registry	Denmark	1995–2011	0.8 million	5–17	Not specified	SSRI	Prevalence and incidence	Number of patients with at least one dispensation in a calendar year divided by the total study population within the same calendar year.	Number of patients with at least one dispensation in a calendar year, and no dispensation during the past 2 years, divided by the total number of person‐years within the same calendar year.
Rasmussen 2024	Nationwide prescription registries	Denmark, Norway, Sweden	2007–2018	2.8 million	5–17	Not specified	All antidepressants	Incidence	NA	Number of patients with at least one dispensation in a calendar year, and no dispensation during the past 2 years, divided by the total number of person‐years within the same calendar year.
Saastamoinen 2012	Nationwide prescription registry	Finland	1999–2005	Not specified	0–17	Not specified	SSRI	Incidence	NA	Number of patients with a new dispensation within a calendar year and no dispensation during the past 1 year, divided by the total study population within the same calendar year.
Sihvo 2008	Population‐based survey linked to nationwide prescription registry	Finland	1999–2003	7112	30 and older	52	All antidepressants	Prevalence	Number of patients with at least one dispensation in a calendar year divided by the total study population within the same calendar year.	NA
Sihvo 2010	Nationwide prescription registry	Finland	1994–2003	4.1 million	18 and older	Not specified	All antidepressants	Prevalence	Number of patients with at least one dispensation in a calendar year divided by the total study population within the same calendar year.	NA
Skovlund 2017	Nationwide prescription registry	Denmark	2000–2013	40.2 million person‐years	10–49	Not specified	All antidepressants, excluding bupropion	Prevalence and incidence	Number of patients with at least one dispensation in a calendar year divided by the total study population within the same calendar year.	Number of patients with at least one dispensation in a calendar year, and no previous dispensation, divided by the total number of person‐years within the same calendar year.
Steffenak 2012	Nationwide prescription registry	Norway	2006–2010	Not specified	15–16	Not specified	All antidepressants	Prevalence	Number of patients with at least one dispensation in a calendar year divided by the total study population within the same calendar year.	NA
Steinhausen 2014	Nationwide prescription registry	Denmark	1996–2010	Not specified	0–17	Not specified	All antidepressants	Prevalence	Number of patients with at least one dispensation in a calendar year divided by the total number of person‐years within the same calendar year.	NA
Wastesson 2012	Nationwide prescription registry	Sweden	2008	0.5 million	80 and older	63.7	All antidepressants	Prevalence	Number of patients with at least one dispensation in a calendar year divided by the total study population within the same calendar year.	NA
Wesselhoeft 2020	Nationwide prescription registries	Denmark, Norway, Sweden	2007–2017	3.7 million	5–17	Not specified	All antidepressants	Prevalence	Number of patients with at least one dispensation in a calendar year divided by the total study population within the same calendar year.	NA
Zito 2006	Population‐based prescription database	Denmark	2000	0.5 million	0–19	Not specified	All antidepressants	Prevalence	Number of patients with at least one dispensation in a calendar year divided by the total study population within the same calendar year.	NA
Zoëga 2009	Nationwide prescription registry	Iceland	2003–2007	Not specified	0–17	Not specified	All antidepressants	Prevalence and incidence	Number of patients with at least one dispensation in a calendar year divided by the total study population within the same calendar year.	Number of patients with at least one dispensation in a calendar year, and no dispensation during the past 1 year, divided by the total study population within the same calendar year.

In the study quality assessment, 29 studies received a score of ≥7, and one study received a score of 5 (Table S2). The individual domains for which almost all studies (*n* = 28) did not score a point were the statistical analysis (due to lack of complete reporting of outcome data) and the description of study participants and setting (*n* = 27).

### 3.3 Prevalence of antidepressant dispensation

The 26 studies on prevalence covered Denmark (*n* = 11, of which 1 also covered Greenland), Finland (*n* = 4), Iceland (*n* = 1), Norway (*n* = 8) and Sweden (*n* = 8) (Table S3).^16^, ^17^, ^18^, ^19^, ^20^, ^21^, ^22^, ^23^, ^24^, ^25^, ^26^, ^27^, ^28^, ^29^, ^30^, ^31^, ^32^, ^33^, ^34^, ^35^, ^36^, ^37^, ^38^, ^39^, ^40^, ^45^ From these, 12 studies reported age‐sex specific prevalences across several years.^17^, ^18^, ^19^, ^20^, ^22^, ^24^, ^28^, ^29^, ^34^, ^35^, ^38^, ^45^

#### 3.3.1 Children and adolescents

**FIGURE 2:**
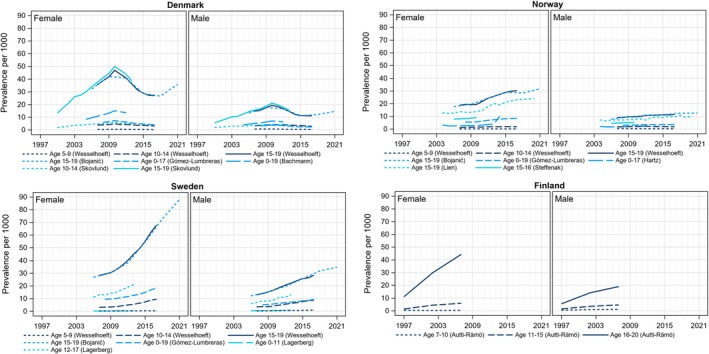
Prevalence of antidepressant dispensation over time by age and sex among children and adolescents in Denmark, Finland, Norway and Sweden. Prevalence is expressed as rate per 1000 population. Presented estimates were selected based on comparable age‐sex groups. Remaining estimates were omitted for clarity as they are described in Table S3.

Overall, the prevalence of antidepressant dispensation was higher among girls than boys and increased with age. Key findings in terms of temporal trends are described below and are illustrated graphically in Figure 2.

In Denmark, estimates between 2000 and 2021 were reported.^18^, ^19^, ^22^, ^34^, ^38^ The prevalence increased until a peak around 2010, after which it began declining. Among girls aged 10–14, the prevalence was 1.7/1000 in 2000 and increased to 3.0/1000 in 2017. Among boys aged 10–14, the prevalence increased from 1.9/1000 to 2.8/1000 during the same period. Among girls aged 15–19, the prevalence increased from 13.1/1000 in 2000 to35.8/1000 in 2021, and among boys of the same age, the prevalence increased from 5.5/1000 to 14.5/1000 during the same period.

In Sweden, estimates between 2006 and 2021 were reported.^19^, ^22^, ^28^, ^38^ The prevalence increased over time with no evidence of levelling off. Among girls aged 10–14, the prevalence increased from 3.0/1000 to 9.4/1000 between 2007 and 2017 and among boys from 3.4/1000 to 8.7/1000. Among girls aged 15–19, the prevalence between 2006 and 2021 increased from 26.8/1000 to 87.9/1000 and among boys from 12.1/1000 to 34.7/1000.

In Norway, estimates between 2004 and 2021 were reported.^19^, ^22^, ^25^, ^29^, ^35^, ^38^ The prevalence among girls aged 15–19 increased from 17.7/1000 to 31.6/1000 between 2006 and 2021 and among boys from 7.8/1000 to 12.5/1000. Among girls aged 10–14 years, the prevalence was 1.1/1000 in 2007 and 1.8/1000 in 2017, and among boys of the same age, it was 1.6/1000 and 1.8/1000, respectively.

Based on the most recent available data, cross‐country comparisons indicated substantially higher prevalence estimates in Sweden than in Denmark and Norway. Among adolescents aged 15–19 years, Swedish girls had 2.46 and 2.78 times higher prevalence (in 2021) compared with their Danish and Norwegian peers, respectively, whereas corresponding estimates among boys were 2.39 and 2.78 times higher. Among children aged 10–14 years, prevalence (in 2017) among Swedish girls was 3.13 times higher than in Denmark and 5.22 times higher than in Norway, whereas Swedish boys had 3.11 and 4.83 times higher prevalence, respectively.

One study from Finland dated further back in time and covered a shorter time frame, reporting prevalence between 1997 and 2007.^17^ Among those aged 11–15, the prevalence in boys increased from 1.5/1000 to 4.6/1000 and in girls from 1.4/1000 to 5.9/1000. Among those aged 16–20, the prevalence in boys increased from 5.5/1000 to 19.0/1000 and in girls from 11.0/1000 to 44.3/1000.

#### 3.3.2 Adults

**FIGURE 3:**
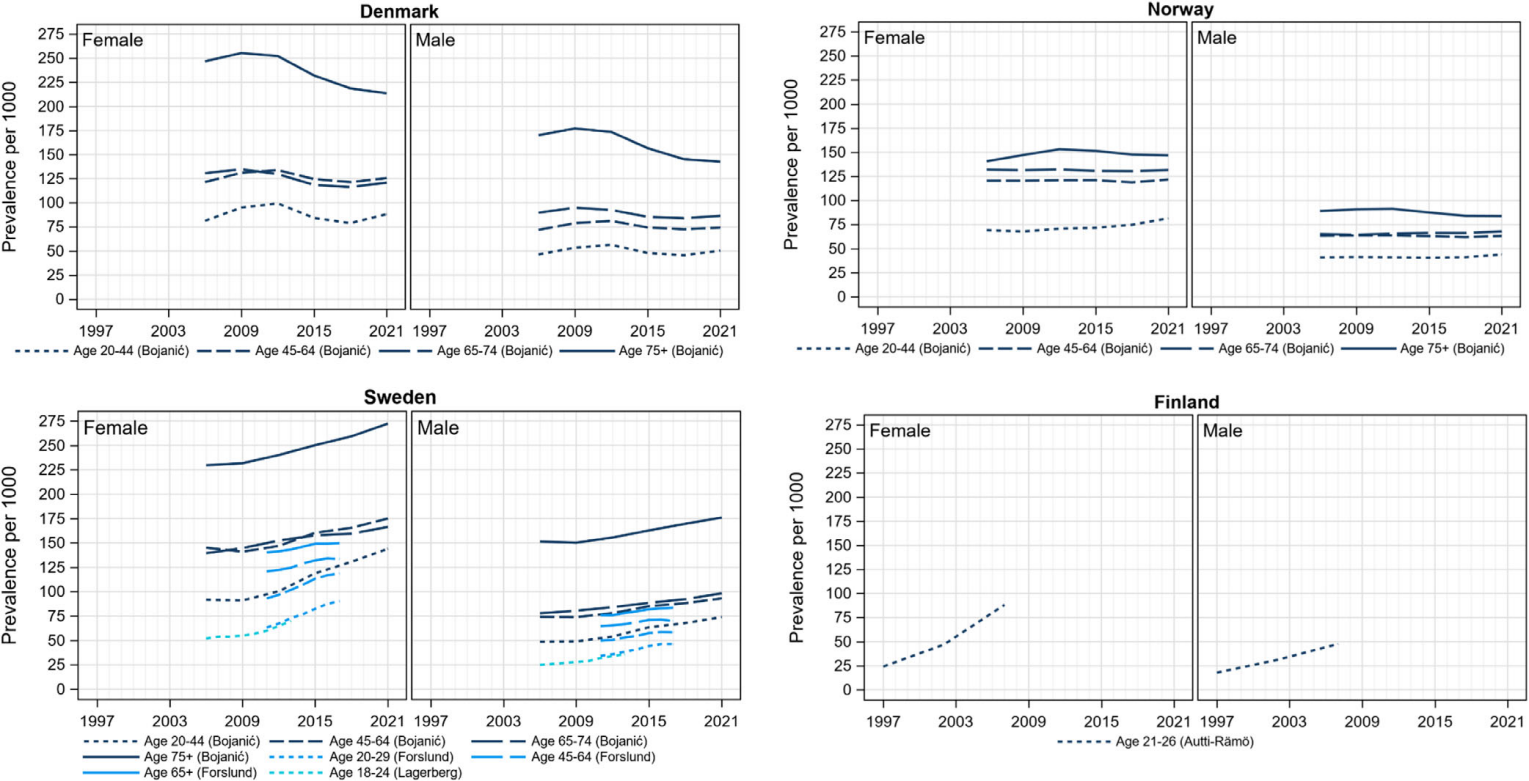
Prevalence of antidepressant dispensation over time by age and sex among adults in Denmark, Finland, Norway and Sweden. Prevalence is expressed as rate per 1000 population. Presented estimates were selected based on comparable age‐sex groups. Remaining estimates were omitted for clarity as they are described in Table S3.

Four of the studies reported age‐sex specific prevalence among adults over time.^17^, ^19^, ^20^, ^28^ Key findings in terms of temporal trends are described below and are illustrated graphically in Figure 3.

In Norway, estimates between 2006 and 2021 were reported.^19^ Among men aged 20–44 years, the prevalence changed from 40.9/1000 in 2006 to 44.0/1000 in 2021, and among women of the same age, the prevalence changed from 69.2/1000 and 81.5/1000 during the same period. For those aged ≥75 years, prevalence changed from 89.0/1000 to 83.8/1000 among men and from 140.6/1000 to 146.8/1000 among women.

In Denmark, estimates between 2006 and 2021 were reported.^19^ Among men aged 20–44 years, the prevalence changed from 46.4/1000 in 2006 to 50.3/1000 in 2021, and among women of the same age, the prevalence changed from 81.3/1000 and 88.2/1000 during the same period. For those aged ≥75 years, prevalence changed from 170.1/1000 to 142.6/1000 among men and from 246.8/1000 to 213.6/1000 among women.

In contrast, the prevalence in Sweden was higher among both men and women across all age groups compared to in Denmark and Norway, and it increased over time.^19^ For example, among women aged 20–44, the prevalence increased from 91.8/1000 in 2006 to 144.1/1000 in 2021 and among men from 48.4/1000 to 74.1/1000. Among those aged ≥75 years, the prevalence among women increased from 229.7/1000 to 272.4/1000 and among men from 151.5/1000 to 176.1/1000.

As such, based on the most recent available data, comparisons across Scandinavian countries showed that Swedish women 20–44 years had 1.63 and 1.77 times higher prevalence compared to their Danish and Norwegian peers, respectively, whereas Swedish men of the same age had 1.47 and 1.68 times higher prevalences. Among those aged 75 years and older, Swedish women had 1.28 and 1.85 times higher prevalence, whereas Swedish men had 1.23 and 2.10 times higher prevalence.

### 3.4 Incidence of antidepressant dispensation

From the 11 studies that reported incidence,^17^, ^21^, ^25^, ^30^, ^31^, ^34^, ^40^, ^41^, ^42^, ^43^, ^44^ we were unable to extract relevant data from one study,^25^ leaving 10 studies covering Denmark (*n* = 4), Finland (*n* = 3), Iceland (*n* = 1), Norway (*n* = 2) and Sweden (*n* = 2) (Table S4).^17^, ^21^, ^30^, ^31^, ^40^, ^41^, ^42^, ^43^, ^44^ Five of these reported age‐sex specific incidences over several years.^17^, ^34^, ^40^, ^41^, ^44^

#### 3.4.1 Children and adolescents

**FIGURE 4:**
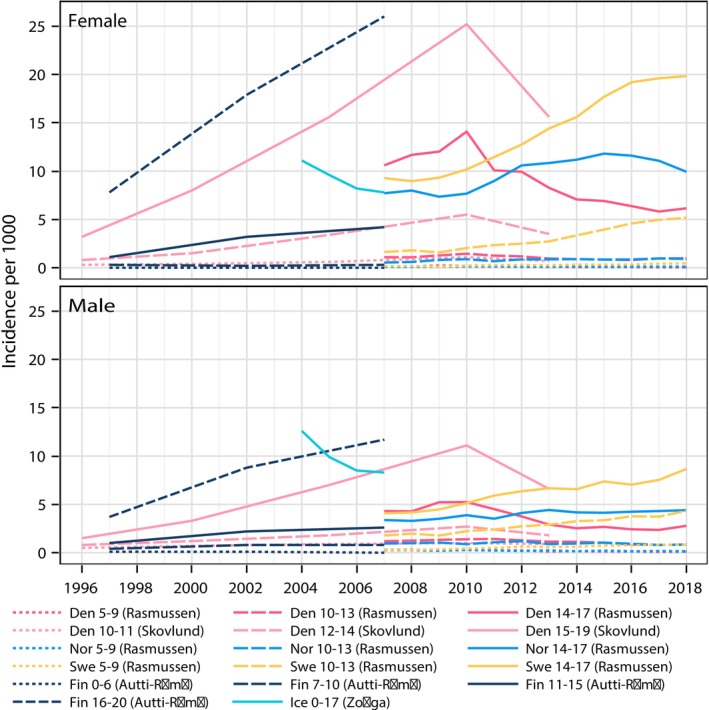
Incidence of antidepressant dispensation over time by age and sex among children and adolescents in Denmark, Finland, Iceland, Norway and Sweden. Incidence is expressed as rate per 1000 population. Presented estimates were selected based on comparable age‐sex groups. Remaining estimates were omitted for clarity as they are described in Table S4.

Key findings in terms of temporal trends are described below and illustrated graphically in Figure 4. One study from Denmark reported on incidence between 1996 and 2013.^34^ The study found that the incidence increased over the study period, particularly among females, although declining from a peak in 2010, similar as for prevalence. For example, among girls aged 15–19, the incidence increased from 3.2/1000 to 15.6/1000 between 1996 and 2013. Another study reported incidences between 2007 and 2018 in Denmark, Norway and Sweden.^41^ Again, in Denmark, the incidence increased during the first few years after 2007 but declined after around 2010. Among girls aged 14–17, the incidence dropped from 14.09/1000 to 6.15/1000 between 2010 and 2018 and from 5.24/1000 to 2.78/1000 among boys. Among girls aged 10–13, the incidence dropped from 1.45/1000 to 0.98/1000 between 2010 and 2018, and among boys, it dropped from 1.42/1000 to 0.85/1000 between 2011 and 2018.

In contrast, incidences in Sweden increased throughout the study period. For example, among girls aged 14–17, the incidence increased from 9.28/1000 to 19.83/1000 4.08/1000. Among boys of the same age, it increased from 4.08/1000 to 8.67/1000. Among those aged 10–13, the incidence increased from 1.63/1000 to 5.17/1000 among girls and from 1.78/1000 to 4.33/1000 among boys.

In Norway, among girls aged 14–17, the incidence changed from 7.71/1000 to 9.93/1000 between 2007 and 2018, although declining from a peak of 11.81/1000 in 2015. Among boys of the same age, the incidence changed from 3.38/1000 to 4.40/1000. Among those aged 10–13, the incidence changed from 0.54 to 0.92/1000 among girls and from 0.95/1000 to 0.83/1000 among boys.

As such, based on the most recent available data (i.e., from 2018), cross‐country comparisons showed substantially higher incidences in Sweden than in Denmark and Norway. Among girls aged 14–17 years, the incidence in Sweden was 3.22 times higher than in Denmark and 2.00 times higher than in Norway. Corresponding figures among boys of the same age were 3.12 and 1.97 times higher, respectively. Among children aged 10–13 years, Swedish girls had 5.28 and 5.62 times higher incidences compared with their Danish and Norwegian counterparts, whereas Swedish boys had 5.09 and 5.22 times higher incidences, respectively.

The studies from Finland and Iceland dated further back in time and covered a shorter time frame. In Finland,^17^ the incidence between 1997 and 2007 increased from 1.0/1000 to 2.6/1000 among boys 11–15 years and from 3.7/1000 to 11.7/1000 among boys 16–20 years. In girls, the incidence increased from 1.1/1000 to 4.2/1000 among those 7–10 years and from 7.8/1000 to 26.0/1000 among those 16–20 years. In Iceland,^40^ the incidence among boys 0–17 years fell from 12.6/1000 in 2004 to 8.3/1000 in 2007 and from 11.1/1000 to 7.8/1000 among girls.

#### 3.4.2 Adults

There were limited data on changes in age‐sex specific incidence in adults (Table S5). In Denmark, the incidence among men and women aged 20–34 increased between 1996 and 2013, with a peak in 2010, whereas among men and women aged 35–49, the incidence in 2013 was roughly similar to or even lower than in 1996.^34^ Another study in Denmark examined the incidence among older adults, reporting a stable incidence between 2016 and 2018 among individuals aged ≥65 years, in both men (27.4/1000 to 25.7/1000) and women (36.8/1000 to 33.4/1000).^44^ In Finland, the incidence increased from 1997 to 2007 in both men (10.8/1000 to 23.8/1000) and women (14.2/1000 to 36.9/1000) aged 21–26 years.^17^

There were no data on trends in the incidence among adults in either Norway or Sweden, with only two studies reporting incidence during a single year.^30^, ^43^ In Norway, the incidence in 2008 among adults 20–39 years was 8.5/1000 in men and 12.7/1000 in women, which is about three times lower than their Finnish peers (i.e., aged 21–26) in 2007 described above.^43^ In Sweden, the incidence in 2010 in the total male population was 15.4/1000 and 24.7/1000 in the female population.^30^

## 4 DISCUSSION

This systematic review confirmed large differences in antidepressant dispensation between the Nordic countries, with a higher and rising level of dispensation in Sweden compared to Denmark and Norway. These differences between countries amplified during later years and were seen across all age groups and both sexes but more pronounced among children and adolescents, and particularly girls.

A key finding of this review is that it revealed a growing divergence in dispensation during recent years, where in Denmark and Norway, there has been either a stable, less pronounced or even declining rate of dispensation, as opposed to a continuous increase in Sweden. A recent review came to similar conclusions but only covered prevalence data until 2017 and incidence data until 2013.^9^ Our review confirms that the gap has further widened in recent years. As such, based on the most recent data, the prevalence of antidepressant dispensation among Swedish 15‐ to 19‐year‐olds was about three times higher, and among 10‐ to 14‐year‐olds up to five times higher, than their Danish and Norwegian peers. The incidence was also about four times higher among Swedish boys and girls aged 10–13 compared to their Danish and Norwegian peers and about two to three times higher among those aged 14–17. Whereas the differences in absolute terms were relatively small among those aged 10–14, the differences were larger among those aged 15–19. For example, the difference in prevalence when comparing Sweden to Denmark and Norway was approximately 52 to 56/1000 among girls and approximately 20 to 22/1000 among boys. Additionally, whereas the previous review focused exclusively on young people, our review suggests that differences between countries are evident also in adults. Although relative differences were smaller than for children and adolescents, there were large absolute differences. For example, among women aged 20–44 years, the difference in prevalence was approximately 56 to 62 per 1000, and among women aged 75 years and older, the difference was approximately 125 to 129 per 1000.

The reason for the shifts and variations in dispensation patterns is likely multifactorial. We cannot determine any causes for this because our review was descriptive in its nature, but factors such as drug reimbursement, licensing and treatment recommendations are unlikely to play a major role in explaining the differences between countries. Denmark, Norway and Sweden have similar drug reimbursement systems,^7^ licensing status of antidepressive medications and treatment guidelines. In recent work, we found substantial similarities between the Swedish Medical Products Agency's treatment recommendations and corresponding national treatment guidelines aimed at prescribers in the other Nordic countries.^46^ For example, psychological treatment was generally recommended for milder cases, whereas pharmacological treatment was often suggested as severity increased. Yet it should be noted that it is challenging to compare treatment guidelines because definitions (e.g., disease severity and presence/absence of diagnosis) vary between countries, and within a country, there may be several different guidelines and different guideline developers (governmental authorities, professional associations, etc.). In any case, the findings of this review shed light on the importance of further analytical studies that seek to address potential causes for the shifts and variations in dispensation patterns, some of which are exemplified below.

One aspect could be to examine whether there are variations between countries in compliance with treatment guidelines. Specifically, it would be interesting to learn whether there may be cross‐country variations in clinician treatment preference and prescribing practices, as well as in patient adherence to pharmacotherapy. These are factors that are not captured by using registry data on dispensed medications. Further, it would be relevant to examine potential differences in the availability and out‐of‐pocket costs of pharmacological *vs*. non‐pharmacological treatment. In Sweden for example, the National Board of Health and Welfare has stated that the availability of psychotherapy in the treatment of depression and anxiety disorders needs to increase.^47^ Further, because the prevalence measure can be challenging to interpret as it is dependent on both the incidence and treatment duration, studies could seek to address whether there have been changes over time in treatment duration in the Nordic countries,^41^ which then could be taken into account when interpreting the differences in prevalence across countries.

It may also be valuable to examine whether there are differences between countries in terms of underlying psychiatric disorders where antidepressant medication is indicated and if this could partly explain the difference in dispensation.^48^, ^49^ A recent multinational study estimated that the prevalence of major depression was 3.1% in Sweden, 3.2% in Denmark and 4.4% in Norway.^5^ A similar pattern is seen in data from the Global Burden of Disease, where the prevalence of depression and anxiety is estimated to 5.1% and 5.9% in Sweden, 4.6% and 6.2% in Denmark and 4.4% and 7.5% in Norway.^8^, ^50^ Other factors that could be considered in future research are more structural ones, such as the role of different care pathways and staff availability. For example, Sweden has been reported to have a higher availability of child and adolescent psychiatric specialists,^51^ and Swedish residents can use self‐referral to specialist care, where the majority of antidepressants to children and adolescents are prescribed.^41^ Thus, it is possible that part of the higher dispensation among young people in Sweden may relate to better access to qualified, specialist care; this needs to be further examined. Finally, although difficult to study, sociocultural factors may be involved. For example, a study from Denmark suggested that the downward trend in incidence from around 2010 was the result of population scepticism towards antidepressant use as a result of increased media attention at this time, mainly revolving around side effects.^52^ Collectively, the findings of this review show that analytical studies are warranted to understand the causes for the observed dispensation patterns both within and between the Nordic countries. This would be important for evaluating equality of care and to ensure alignment with evidence‐based practice. These insights may ultimately inform safer prescribing, more efficient resource allocation and improved mental health care.

### 4.1 Limitations and strengths

This work has some limitations that should be considered. First, we did not search grey literature for potential articles, which means that we cannot rule out a risk of publication bias in this review. At the same time, this approach likely reduced the risk of including low‐quality studies. Second, there were not studies from all the Nordic countries reporting estimates from recent years within comparable age‐sex strata to justify a meta‐analysis. Given the large shifts in antidepressant dispensation over time, pooling earlier studies with later studies would likely introduce major heterogeneity. In any case, this does not affect the implications of this review because the focus was to describe variations *between* countries and not the overall level of dispensation. Third, our review showed that there were much less data on antidepressant dispensation in adults compared to in children and adolescents, particularly regarding incidence, and this should be the target of future studies. Fourth, recent data on antidepressant dispensation in Finland and Iceland were lacking, and we only identified a very small number of studies with largely historical data. This prohibited us from performing comparisons with these countries. By implication, even though the rates were higher in Sweden compared to Denmark and Norway, this should not be interpreted as if Sweden has the higher rates out of all Nordic countries. We therefore urge researchers to conduct and publish up‐to‐date analyses of antidepressant dispensation in these countries. Ideally, a large, multinational study including data from all Nordic countries and with harmonized definitions would be highly valuable. Fifth, because our review was restricted to studies conducted in the general population, and without data on the indication for treatment, the findings may not be generalized to certain segments of the population. Thus, it is possible that the temporal trends may differ between subgroups, such as depending on socioeconomic factors and comorbidities, and this should be further investigated. Sixth, although a proxy of antidepressant drug consumption, prescription data from the Nordic registries only include information on drugs dispensed at pharmacies. This neither tells us whether and to what extent patients actually consumed the drugs. Seventh, the quality of included studies was overall rated as high, as based on the Joanna Briggs Institute checklist. However, many studies often did not report all the necessary data from their statistical analysis, leading to a no‐response on this domain. This does not imply that the studies were poorly analysed, but rather, it stresses the importance of complete and transparent reporting of all necessary data but also that the checklist may lack granularity. Eighth, there were some variations between studies in terms of the wash‐out periods applied in the analyses of incidence, which can influence the results. However, the most recent study on age‐sex‐specific trends was a multinational study covering Denmark, Norway and Sweden, where the same wash‐out period was applied across countries, and this is where most of our inferences were made regarding incidence differences in youth. Ninth, although two reviewers independently selected studies, extracted data and rated study quality, we did not perform inter‐rater reliability tests.

Strengths of this review include systematic searches by an information specialist in three databases, the inclusion of about threefold the number of studies compared to the most recent similar review, the inclusion of studies on both prevalence and incidence across all ages, and the detailed breakdown of the data compared to the previous review, allowing for clearer insights into subgroup differences.

## 5 CONCLUSIONS

In summary, this systematic review highlights a growing divergence in antidepressant dispensation between the Nordic countries, particularly in children and adolescents, and especially girls. These findings underscore the need for analytical studies examining factors underlying temporal trends and cross‐national differences in antidepressant dispensation, including incidence trends among adults, as well as updated population‐based studies from Finland and Iceland to enable comprehensive Nordic comparisons.

## AUTHOR CONTRIBUTIONS

All authors contributed to the conception and/or design of the study. Marcel Ballin, Mats Talbäck and Paulina Tuvendal contributed to study selection, data extraction and quality assessment. Marcel Ballin created the tables and drafted the manuscript. Mats Talbäck created the figures. Rickard Ljung provided supervision throughout the study. All authors interpreted the data. All authors critically revised the manuscript for intellectual content. All authors reviewed and approved the manuscript for submission.

## CONFLICT OF INTEREST STATEMENT

The authors declare no conflicts of interest.

## Supporting information


**Table S1.** Excluded studies and reasons for exclusion.

**Table S2.** Study quality in individual studies according to the Joanna Briggs Institute Critical Appraisal Checklist for Studies Reporting Prevalence Data.

**Table S3.** Results in studies on prevalence of antidepressant dispensation.

**Table S4.** Results in studies on incidence of antidepressant dispensation.

**Table S5.** Domains of reported prevalence and incidence data by country.

**Data S1.** Supporting Information.

**Data S2.** Supporting Information.

## Data Availability

The data presented in this study were extracted from previously published studies, which are cited in the reference list. No new primary data were generated.
