# Supplementary material for: Trends in age‐sex‐specific prevalence and incidence of antidepressant dispensation in the Nordic countries: a systematic review
Source: Br J Clin Pharmacol. 2026 Mar 24;92(7):2049–62. doi: 10.1002/bcp.70540 (PMC13304260; doi:10.1002/bcp.70540)
Supplement: Supplementary file 5 — Table S5. Domains of reported prevalence and incidence data by country. [file BCP-92-2049-s006.docx]

| **Supplemental table 5.** Domains of reported prevalence and incidence data by country. | | | | | |
| --- | --- | --- | --- | --- | --- |
|  | **Country** | | | | |
|  | **Denmark** | **Finland** | **Iceland** | **Norway** | **Sweden** |
| **Children and adolescents** |  |  |  |  |  |
| ***Prevalence*** |  |  |  |  |  |
| Year of most recent   data (age group) | 2017 (5–14 years)  2021 (15–19 years) | 2007 (0–20 years) | 2007 (0–17 years) | 2017 (5–14 years)  2021 (15–19 years) | 2017 (5–14 years)  2021 (15–19 years) |
| Stratified by sex? | Yes | Yes | No | Yes | Yes |
| ***Incidence*** |  |  |  |  |  |
| Year of most recent   data (age group) | 2018 (5–17 years) | 2007 (0–20 years) | 2007 (0–17 years) | 2018 (5–17 years) | 2018 (5–17 years) |
| Stratified   by age and   sex? | Yes | Yes | Yes | Yes | Yes |
| **Adults** |  |  |  |  |  |
| ***Prevalence*** |  |  |  |  |  |
| Year of most recent   data (age group) | 2021 (20 years and older) | 2007 (21–26 years) | - | 2021 (20 and older) | 2021 (20 and older) |
| Stratified   by age and   sex? | Yes | Yes |  | Yes | Yes |
| ***Incidence*** |  |  |  |  |  |
| Year of most recent   data (age group) | 2013 (20–49 years)  2018 (65 years and older) | 2007 (21–26 years) | - | 2008 (20 years and older) | 2010 (all ages) |
| Stratified   by age and   sex? | Yes | Yes |  | Yes | Yes |
